# Early Development of Chinese Sign Language in Shanghai Schools for the Deaf

**DOI:** 10.3389/fpsyg.2021.702620

**Published:** 2021-07-01

**Authors:** Hao Lin

**Affiliations:** Institute of Linguistics, Shanghai International Studies University, Shanghai, China

**Keywords:** Chinese sign language, Shanghai variant, media of instruction, early development, deaf school, early language

## Abstract

The Shanghai variant of Chinese Sign Language (SCSL) is one of the main variants of Chinese sign languages, greatly influencing other sign languages, such as Hong Kong Sign Language and Singapore Sign Language. This paper is a first attempt to trace its origins and early history and deaf education in Shanghai until 1949. The data are collected in two ways: first, by delving into the archives, i.e., written records of deaf history and education in China during that time; second, by interviewing surviving deaf students who went to school before or around 1949. Our findings are as follows: (a) SCSL began in the 1920s and emerged as a distinct sign language in the 1940s. Two deaf schools were the power shaping its progress among several deaf schools established in Shanghai: Fryer deaf school and Group learning deaf school. The sign variants of these two schools form the backbone of SCSL. (b) Deaf teachers are one of the key factors that affect the early development of a sign language. Chinese deaf played a vital role in the rise and spread of SCSL in the 1930s and 1940s, as some deaf teachers opened deaf schools in Shanghai and other cities, even other countries or areas, thus helping SCSL to spread. (c) Arising in an international and multilingual environment, SCSL is characterized by traces of foreign sign languages, especially ASL, due to language contacts linked to deaf education at that time, e.g., some proper names, like XUJIAHUI, SHANGHAI-1 and some high-frequency words like water. (d) However, foreign sign languages' direct influence is negligible due to the lack of participation of deaf foreigners in deaf education in Shanghai and oralism advocated by foreign educators in relevant deaf schools. To sum up, deaf teachers for deaf schools are key to the early development and spread of SCSL.

## Introduction

Shanghai Sign Language or, to be more precise, the Shanghai variety of Chinese Sign Language (SCSL) has been regarded as prototypical of southern CSL (here, CSL is used as a term covering all varieties of sign languages in China), one of two major varieties of CSL (Fischer and Gong, 2010). Historically, it has had an impact not only on other areas in the China mainland, but also on other sign languages overseas, such as Macau Sign Language, Hong Kong Sign Language (Sze et al., 2013), Taiwan Sign Language (TSL, Tai and Tsay, 2015) and Singapore Sign Language. Its status is prominent as one of the key factors in deaf education in China. Currently, SCSL is the mother tongue of about 174,700 Shanghai deaf people, which excludes the hard-of-hearing (Ni, 2007). However, now, SCSL seems to be endangered due to a series of factors, such as a decrease in new signers and pressure from Chinese Sign Language standardization supported by national language policy. It is important and urgent to explore how it emerged as its history is not long, and some old deaf who witnessed its emergence still are living.

Generally, a deaf community's existence is the fundamental condition based on which a sign language emerges and evolves, generation after generation. The cities offered a chance for the deaf to gather together and the conditions for establishing deaf schools. In modern times, deaf schools provide a natural space for deaf people to gather and interact intensively. For example, Senghas et al. (2005) describe the emergence of Nicaragua Sign Language. Before the 1970s, it was supposed that there was no deaf community, deaf people lived isolated lives and mainly used home signs and gestures for daily communication. The opening of a public deaf school in 1977, which was followed by another deaf school nearby in 1980, brought together several hundred deaf students in the early 1980s. They argued that deaf children spontaneously developed Nicaragua Sign Language in the school with scarcely any language input. In no more than 20 years, it evolved from a creole system used by an older generation of students to “a fully-fledged, primary sign language, resulting from the process of nativization,” as Senghas (1995, p. 1) puts it. On a broader scale, as language practice in a deaf education context typically has a great impact on sign language, the history of American Sign Language shows that deaf teachers often played a key role in transmitting sign languages (Nover, 2000; Reagan, 2010). It was the French deaf teacher Laurent Clerc (1785–1869) who laid the foundations of ASL and was the main driver of the LSF (French Sign Language) element in ASL, as he worked in the American School for the Deaf (ASD) as the first deaf teacher and was a mentor in that school for many years. Through his students and deaf colleagues, the early variety of sign language used in ASD was transmitted all across the U.S.A. (Nover, 2000; Reagan, 2010).

Similarly, the generic relation between European sign languages is largely determined by the above two factors: deaf schools and deaf teachers. The sign languages of Germany, Austria, and Hungary belong to the same family, as teachers of the deaf were trained in Austria, and they influenced deaf communities (Brentari, 2010). The British Empire spread British Sign Language to its colonies, like Australia and New Zealand, so Auslan and NZSL are closely related to BSL (Woll et al., 2001).

To date, little research has discussed the history of Chinese Sign language and its relationship to other attested sign languages with possible historical connection. We may expect something similar to SCSL, like key deaf figures and important deaf schools. What happened during its early development, and how did it happen? This paper mainly aims to trace the origins and development of SCSL based on an exploration of these deaf schools and their deaf students and teachers. We will first present a brief introduction to the social context for the early development of SCSL. In Section Research Questions and Methodology, we will introduce the research questions and the methodology. Section Data Analysis analyzes the major deaf schools and important figures in Shanghai deaf education and their interaction, it also examines in depth how sign languages were implemented in educational activities and practiced in deaf people's daily lives. This led to the evolution of the present form of Shanghai Sign Language. In section Discussion, we will talk about its implications in relation to the early development of sign languages in general. Finally, the conclusion will summarize our findings.

### Social Context for the Early Development of SCSL

First, we will trace Shanghai's change from a town in 1842 to a metropolis with a population of more than 5.4 million in 1949. In the unequal treaty of Nanjing in 1842, Shanghai was forced to become a port open to foreigners. The British, French, and Americans managed to acquire concessions (a de facto enclave) just outside the old town from the Qing Dynasty in 1854, which mainly consisted of two concessions: a French concession and an international settlement, the latter being mainly shared by British and Americans. Though some other nations also joined in, it was controlled by the British. Unlike other areas of Shanghai, these concessions were governed by respective foreigners and subject to foreign laws. These concessions became the center of Shanghai as time went by, where the Chinese community and foreigners mixed after 1854. With a growing economy and a comparatively peaceful situation, Shanghai concessions attracted several surges of immigration that brought Chinese from other places and large populations of foreigners from around the world, who were attracted by the economic opportunities, comparative stability, and tolerance. The first leap from a town to a middle-size city took place in the 1840s−1850s, when Shanghai replaced Guangzhou as the biggest trade port. However, it was not until the 1890s that Shanghai became a center for trade, finance and manufacture. And the population grew very rapidly, sometimes doubling every 15 years, from 1880 onwards. In the concessions, the population of foreigners increased from only 26 in 1843 to 15,012 in 1910, 59,285 in 1930, and peaked at more than 150,000 in around 1940 (Zhang, 2001) (see Table 1).

**Table 1:** Increase of the population in Shanghai concessions (thousands) Zhang (2001).

**Year**	**1880**	**1895**	**1910**	**1922**	**1932**	**1937**	**1940**	**1949**
Population	140	290	610	1,000	1,500	1,700	4,000	5,400

It features high density in the population and heterogeneity among its residents. As for the Chinese, local Shanghai people (born in Shanghai) accounted for only 15 to 22 from 1885 to 1930, while the majority were immigrants from other places. More than half of the immigrants came from Jiangsu province, and one third moved from Zhejiang province. Similarly, there were 150,931 foreigners in 1942 from about 51 countries. The British topped the list before 1915 and were then replaced by the Japanese. Besides, large numbers of Russian and Jewish refugees lived in Shanghai between the two World Wars because Shanghai was one of few places that received Jewish refugees and the only place that anyone in the world could settle without requiring a visa (Shanghai Local History Committee, 2005). Shanghai's social context offered unique conditions for SCSL's early development: a comparably peaceful and civilized place for the wealthy, a migrant city, and a cosmopolitan city that was tolerant of multiculturalism and multilingualism (Brizay, 2010).

There are few recordings of deaf activities in Shanghai in the Nineteenth century. We could not find any census details of deaf residents before 1949, due to the ignorance of the local establishment during chaotic periods between the end of the Nineteenth century and 1949. However, there were probably deaf people living in or nearby the concessions, given the high density and large population, starting no later than the 1880s. As a case in point, French missionaries started an orphanage in 1855, which moved to Xujiahui in 1869. Some of the orphans were abandoned deaf children. News about the deaf appeared sporadically in Shanghai media from the 1900s. In 1919, certain deaf schools' news report began to appear in Shen Bao^1^, followed by a couple of similar news report in the 1920s. In a piece of sensational news reported in 1929, a deaf boy eloped with a hearing girl, was sued by the girl's mother, and put into prison^2^. The 1930s saw a surge in the immigrant population in Shanghai. It also suggests an enlargement of the Shanghai deaf community and saw the establishment of the Zhonghua Deaf Association in 1937, which highlighted the social power of deaf people in Shanghai^3^. It suggests the size of the deaf community, since its members were reported to number one thousand (Lu, 2012). It appeared to be a special international deaf community, which is confirmed by the memoir of Song (2000, p. 98–99):

“(In about the year 1949) not only Chinese deaf, but also some foreign deaf friends frequented the Zhonghua Deaf Association. At first, we were puzzled by their sign languages, but as time went by, they quickly picked up our local signs and could sign the same way as we do. When I look back, I remember that they were foreign deaf, both males and females. The majority of them seemed to be Russians and Jews … Zhang Zhiwan (one of his deaf friends) married a Russian deaf girl who signed perfect Shanghai Local Sign Language. I guessed her Russian Sign Language had been assimilated while living in China, especially after marriage” [p. 80, direct translation from Chinese, Song (2000)].

It appears that a deaf community with strong connections and frequent interactions both internally and externally came into being at the end of the 1940s, though it is hard to estimate how large it was.

### The Development of Deaf Schools in Shanghai, a Short Introduction

When we try to trace the history of deaf education in Shanghai, we also have to turn to the Western missionaries. From the 1890s to the 1920s, there was a great surge of church schools in China, as it was a period of chaos when the central government was weak and warlords across China fought one another. Meanwhile, all schools of Christianity prospered and were enthusiastic about taking over the responsibility of educating local people. The educational establishments ranged from the highest level, like universities, to primary schools at the lowest level. A few priests devoted themselves to the education of the disabled.

#### Xujiahui Deaf School, the First Deaf School in Shanghai

As the central city for foreigners from the second half of the Nineteenth century, Shanghai was chosen as an ideal place for church-sponsored education. French Jesuits started an orphanage in 1864 in Xujiahui (literally “Xu family junction,” named after Xu Guangqi, a high-ranking official in the Ming Dynasty (1368–1644), who was one of the most famous Chinese literati converts baptized by French Jesuits, and settled his family there in the Seventeenth century). Xujiahui developed into the center of the French concession at the end of the Nineteenth century. It was one of the most prosperous areas in Shanghai at that time. The French missionaries founded Xujiahui Cathedral and other religious buildings like St. Ignatius Convent there, and French missionaries ran various charities in these places, including opening a girls' schools. In about 1892, as the Jesuits found more deaf children in the orphanages and they needed to be educated separately from hearing orphans, and so the nuns of St. Ignatius Convent founded a deaf school for those deaf orphans, which was later known as the Xujiahui deaf school. This marked the start of deaf education in Shanghai. Xujiahui deaf school was esoteric due to the strict religious management by French Catholics at the beginning, so it was much less known by outsiders, except for those living nearby or in the Catholic system. It is the only deaf school established by Catholics in China.

#### Other Shanghai Deaf Schools, in Order

Following the Xujiahui deaf school of St. Ignatius Convent, other deaf schools opened in Shanghai in different periods, with different sponsors, active from the 1920s to the 1940s, despite poverty and wars. They are as follows:

a. The deaf-mute school affiliated to the Group Learning Society (Qun Xue Hui Long Ya Xue Xiao^4^)
b. Wide Love deaf-mute school (Hui Ai Long Ya Xue Xiao)
c. Fryer deaf school (Fu Ya Long Ya Xue Xiao)
d. Shanghai private deaf school (Shanghai Si Li Long Ya Xue Xiao)
e. The deaf-mute school affiliated to the Zhonghua Deaf Association (ZhongHua Long Ya Xue Xiao)
f. Shanghai Deaf Youth (Shanghai Ya Qing Xue Xiao)
g. Guangzhen deaf school (Guang Zhen Long Xiao)
h. SongJiang deaf school (Song Jiang Long Xiao).

They will be introduced by their classification into two types: those set up by local Chinese, those set up by priests or foreigners.

(1) Shanghai deaf schools set up by the locals

The deaf school affiliated to the Group Learning Society (henceforth Group Learning deaf) was founded by the Group Learning Society, which was an influential social club (1904–1953) in Shanghai (Zhu, 2011; Lu, 2012). Its members were mainly educationists and philanthropists focusing on the mission of education. As one of the earliest deaf schools set up by local Chinese, it lasted for 17 years and closed in 1938 due to the Japanese occupation of Shanghai and other factors like the headmaster's death. This school is distinct because it was based on the ideals of the Chinese and the first deaf school free of any religious background. It recruited many students and was the main trainer of teachers of the deaf for other deaf schools in the 1930s. It laid the foundations for the emergence of SCSL. For example, Shanghai Private Deaf School (1933–1955) was an offspring of Group Learning Deaf and its founder, Shi Dianqing. Shi was initially a hearing teacher at the Group Learning school. He then left and opened the Shanghai Private Deaf School. The Zhonghua deaf school, affiliated to the Zhonghua Deaf Association, was opened in 1937 by a deaf student and later a teacher from Group Learning Deaf, He Yulin, chairman of the Zhonghua Deaf Association (ZDA). It played an essential role in educating deaf students and deaf refugees, who came from the local area and other parts of China during wartime (1937–1945 Sino-Japan War and 1946–1949 civil war). From 1938 to the end of 1940, another three deaf schools were opened. The first one was the Shanghai Deaf Youth School (henceforth Deaf Youth), founded in 1938 by Hu Wenyi, a student from Shanghai Private Deaf. The second one was the Guangzhen Deaf School (henceforth Guangzhen), set up in 1942 by a couple (the husband was a deaf teacher, the wife was a hearing teacher) who both taught in Zhonghua Deaf as colleagues. The third one established before 1949 was the Songjiang Deaf School, which was opened in 1946 by a deaf teacher who used to work at Fryer School.

(2) Foreigner-initiated Shanghai deaf schools

Another lineage of deaf schools consisted of the Wide Love Deaf School and the Fryer Deaf school. Wide Love Deaf was founded by a Chinese Presbyterian, who later transferred his deaf students to Fryer Deaf School, founded in 1926 by John Fryer (1839–1928), a British priest who spent half his life mainly working for the Qing Dynasty as a translator and educator. He established the deaf school named after him and founded the Fryer Blind School in Shanghai (1912–1955)^5^. After establishing the deaf school, he mainly stayed in America, and its management was entrusted to his son, George B. Fryer (Fu Bulan in Chinese), who was the president. However, Mr. Fu was mainly occupied with the blind school. The deaf school was managed by the vice president, Wang Chongjing, a Chinese hard-of-hearing. Fryer and the committee board's fame, along with Fryers and Mr. Wang's excellent management, made the Fryer Deaf School distinguished among the deaf schools in Shanghai regarding its quality; it also charged higher tuition fees than other deaf schools. After its opening, it was seen as prestigious and attracted quite a few deaf students from wealthy families. It is estimated that over 200 students graduated from it. Some of the deaf students picked deaf education as their profession, either by opening a deaf school or becoming a teacher in deaf education; for example, Dai mu opened a deaf school in his home town, Changzhou, in 1944, and later became a leader in deaf education, as he was appointed chairman of the China Association of the Deaf and Hard-of-hearing. Table 2 profiles these deaf schools, showing their duration, founders, and successors after the establishment of the People's Republic of China (PRC).

**Table 2:** A list of deaf schools before 1949.

**No**.	**Deaf school**	**Duration**	**Founder(s)**	**Successor (if any)**
1	Xujiahui	1892–1952	French priestesses	Closed
2	Group learning	1920–1937	Gao Shoutian	Closed
3	Wide love	1922–1926	Yu zhongzhou	Fryer
4	Fryer	1926–1953	John Fryer	Deaf 2
5	Shanghai Private	1933–1955	Shi Dianqing	Deaf 4
6	Zhonghua	1937–1955	He Yulin	Deaf 4
7	Deaf Youth	1938–1956	Hu Wenyi	Deaf 3
8	Guang zhen	1944–1953	Li Dingqing	Deaf 1
9	Songjiang	1946–1952	Dai Binglong	Closed

*Please also refer to Figure 5A*.

There were two consequences: (1) the old deaf teachers were sidelined and faded away with time, and hearing teachers, newly trained for the deaf schools, began to dominate Shanghai deaf schools from the late 1950s; (2) Shanghai Sign Language was ostracized or at least marginalized in the deaf educational context. It was further compounded by national language policy, and manual alphabets meant to represent *pinyin* started to find its way into it (Zhou, 1964; Sun and Liu, 2007; Gu, 2017). This paper mainly focuses on the time before the foundation of the PRC.

To sum up, we have explored the social context of Shanghai from the 1880s to 1949. In half a century, Shanghai's progress provided the conditions for the deaf to gather together, i.e., it gave birth to a large, open deaf community. Meanwhile, deaf education began to flourish thanks to three groups: foreign missionaries, represented by Xujiahui Deaf and Fryer Deaf, local societies represented by Group Learning Deaf, and the deaf educators themselves, represented by Zhonghua Deaf. We need to investigate how SCSL came into being in these deaf schools, the driving forces for shaping it, and how it spread to other places, even overseas.

## Research Questions and Methodology

Accordingly, we have three research questions:

- When did SCSL start and develop in deaf schools?
- How were sign languages practiced in these deaf schools, respectively, and what was their relation and effects?
- What are the teaching methods in these deaf schools?
- What were the shaping powers in the early development of SL in different phases?

As for methodology, as the literature of history of deaf education is ignored or scattered before 1949, it is very hard to string together fragmentary information and guarantee its validity only via secondhand data. To support the research, we went to great lengths to conduct an investigation of old deaf people for a year. As Lucas et al. (2001) point out, school entry is one of the key variables in investigating the sociolinguistics of sign language. The interview group consists of one hearing researcher (Lin Hao, age 38, working for 8 years with the Shanghai deaf community) and two deaf experts, Yang Zaisheng, who is a retired teacher from a deaf school and has strong and wide liaisons with the elderly deaf community (age 67, born deaf, an enthusiastic advocate of the deaf's right to sign language), and Ni Yinjie (35, she was born deaf and comes from a deaf family; therefore, she is also strongly connected to the elderly deaf community). As an effective methodology to look into sociolinguistic issues (Shaw et al., 2015), we adopt ethnography to conduct our research, as it helps us to take full advantage of our subjects. There were only a few elderly subjects available for interview. Such an investigation is both valuable and urgent, as these elderly deaf signers who went to school before 1949 are dying away as a generation. We interviewed eight subjects who went to deaf schools before or around 1949, among which four were over 90 years old (2 females, mean age = 87.8).

This paper's investigation was conducted in the following steps: (1) 5 min warm-up, (2) a questionnaire survey to elicit some key information about SCSL before 1949 and the specific practices of deaf education in deaf schools. ThisI may last for 20–30 min; (3) free conversation, either monolog or dialogue with our deaf interviewers. Sometimes, Step 2 and Step 3 merged together.

To know if there is any chance to identify the old signs, we customized a questionnaire and a vocabulary list for each interviewee. For example, Luo and Chen were asked to sign the basic vocabulary of the French sign language they learned in Xujiahuii Deaf School and some proper names: place names and landmarks in the 1940s to see if there was any trace of language contact between French sign language and local SCSL at that time. Table 3 provides basic information about the interviewees. As we can see, except for Mr. Zhuang, who is the youngest among the interviewees and went to school in 1950, all the other five spent their time mainly in deaf schools before 1949. Besides, deaf students tended to go to more than one deaf school for study or work, except for Luo and Chen, who had all their schooling in Xujiahui Deaf School.

**Table 3:** Major interviewees who attended deaf schools (their ages were recorded at the time we interviewed them).

**Surname**	**Gender**	**Age**	**School and time in deaf school(s)**
Shen	Male	94	Student in Fryer Deaf from 1934 to 1942
Dai	Male	93	Student in Shanghai Private from 1935 to 1937, and Fryer Deaf from 1939 to 1941
Song	Male	96	Student in Shanghai Private from 1935 to1936, and taught in Zhonghua from 1941 to 1950
Peng	Male	92	Student in Hongkong zhenduo^*a*^ from 1937 to 1941; studied and then worked in Zhonghua as a teaching assistant from 1942 to 1946, opened the first deaf school in Singapore in 1953, taking SCSL to Singapore
Luo	Female	86	Student in Xujiahui deaf from 1940 to 1947, later worked there from 1948 to 1952
Chen	Female	83	Student in Xujiahui deaf from 1941 to 191952
Ding	Male	82	Student in Zhonghua deaf school
Zhuang	Male	76	Student in SongJiang from 1949 to 1954 and First Deaf from 1955 to 1957

a *Hong Kong Zhenduo was the first deaf school in Hongkong, it was established by two sisters sponsored by Cheefoo Deaf School Sun and Liu, 2007; Sze et al., 2013*.

### Data Annotation, Coding, and Software

**Figure 1:**
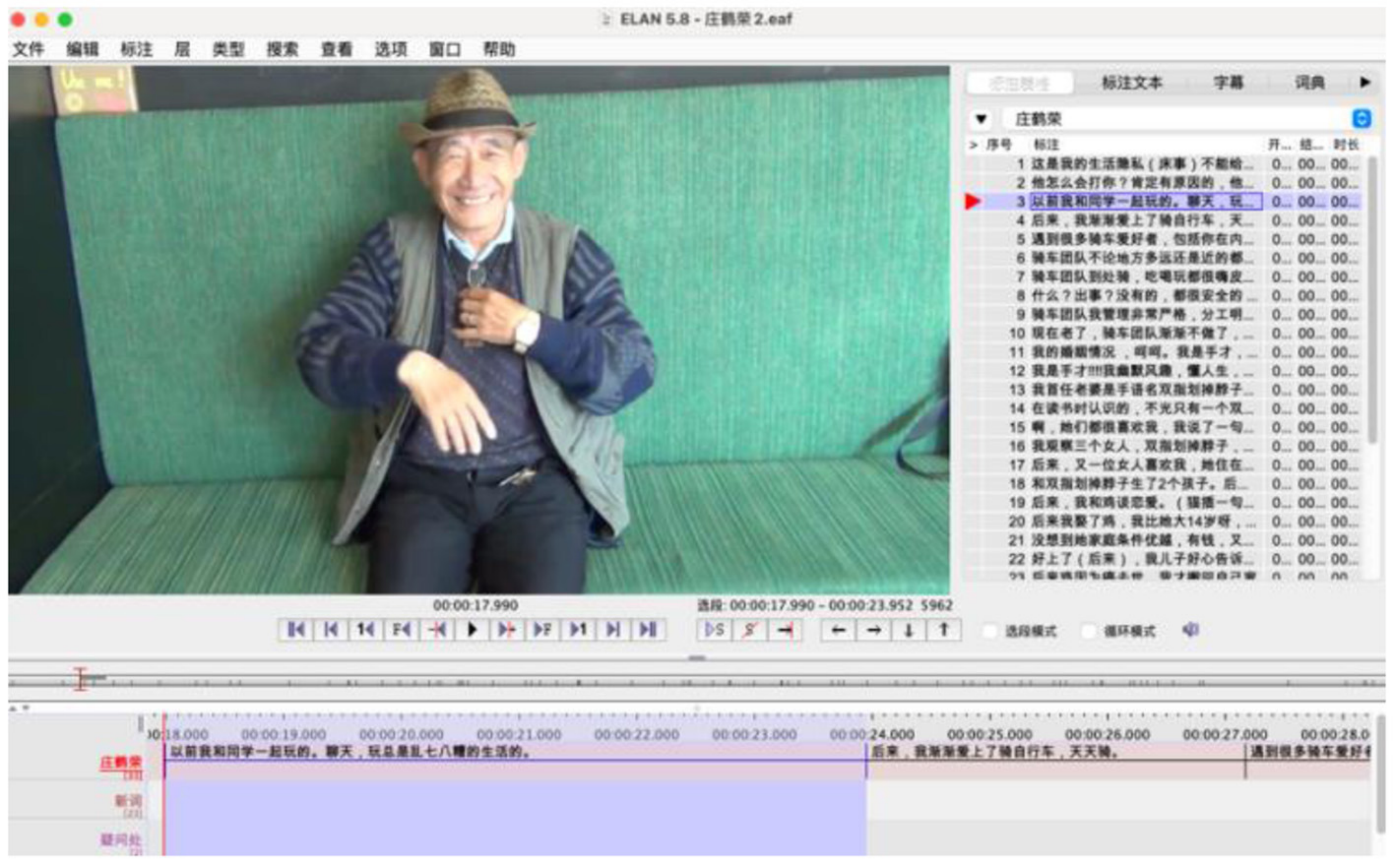
Snapshot of annotated data.

Because there are no existing written words specific to Chinese sign language, we have to rely on Chinese characters in our annotation. Furthermore, as signed translation is based on a visual-spatial dimension, all of our data are preserved in videos (322 min in total). The annotation tool ELAN (https://archive.mpi.nl/tla/elan) is used to annotate the data, as shown in Figure 1. Ms. Ni is responsible for all the annotations, and we checked their accuracy together. All the data in this paper are drawn from annotated data if not explained otherwise. Refer to Appendix 1 for relevant questionaire.

## Data Analysis

It seems that deaf education in Shanghai from 1892 to 1949 can be divided into three phases: Phase 1 (1892–1919), Phase 2 (1920-1937), and Phase 3 (1938–1949). These three phases are marked with distinct features regarding the size of deaf schools, their founders, their staff and, above all, their media of instruction. We will go into the details of Xujiahui Deaf in Phase 1, Fryer Deaf and Group learning in Phase 2, and Zhonghua deaf in Phase 3, to look at how sign language was practiced in these schools. There are three media of communication in deaf education: writing on blackboards, finger-spelling of words, or sign language. We will look at the actual practices of either finger-spelling or sign language in the different schools.

### Phase 1 as Represented by Xujiahui Deaf

Interviewees Luo and Chen were both graduates. Luo was adopted as an orphan and stayed in Xujiahui Deaf from 1938 until its closure in 1952. And Chen was sent to Xujiahui by her family at 8 years old in 1940 and finished her 7-year education there, later working there from 1949 to 1952.

Xujiahui Deaf School was the first deaf school in Shanghai. It was forced to close in 1952 with the French nuns being expelled and the Chinese nuns dismissed due to their Catholic background. The house of St. Ignatius Convent has now been turned into a restaurant. Chen and Luo informed that it was initially founded for deaf female orphans when the church found it was problematic to mix deaf children with hearing students in the same class. Located inside the Catholic building, it was supervised as an esoteric organization with strict rules for the students. Most deaf students were deaf girls, though a few deaf boys before puberty were also educated separately (Chen, 2000).

Some textbooks translated from French deaf schools were used, and French deaf schools' teaching methods were adopted. Chinese nuns were responsible for teaching spoken Chinese by using basic French sign language and French finger-spelling in combination. Basic common words, like *table, book*, and *family*, were learned through practicing their pronunciation. Subjects were Mandarin, mathematics, painting and physics, together with some religious courses. Later it became well-known in the local community, and deaf children from wealthy families were also accepted on condition that they could afford high tuition fees. Signed French with Chinese pronunciation and French finger-spelling were not only the dominant media of instruction in the classroom but the only permitted media for communication in their lives. However, SCSL found its way into this closed world as it started to recruit some deaf children from non-religious families who already knew local sign language. Deaf children in the school began to pick up SCSL from these non-religious children, and SCSL was secretly acquired and practiced by deaf students.

“Chinese pronunciation was taught by touching the necks of oneself and the teacher. We practiced pronunciation by puffing out candles. The pronunciation of everyday Chinese was taught first, for example, *mother, father*. Besides, we spent a lot of time reading the Bible. Every morning we had Mass from 6 to 7 a.m., which was very demanding for us children. Life in school was boring.” (Chen)

“I could use signed French finger-spelling quite well at school, and I managed to learn to pronounce some basic Chinese words, and that is all I could get, so I hated learning Chinese, and preferred the practical subjects, like sewing and drawing. Later, with more students from outside, who could sign, this was the first time I was in touch with sign language and I was quite surprised, because I spoke and they signed when we communicated. It was difficult at the beginning. As time passed, I picked up their signs naturally, which later occupied my mind, and meanwhile my oral Chinese was corrupted day by day. And when I entered society, my living circle was all about deaf people, and sign language become my dominant language, my spoken Chinese and French-spelling almost completely forgotten. However, let me think, I can still remember how to pronounce some basic words.” (Luo)

**Figure 2:**
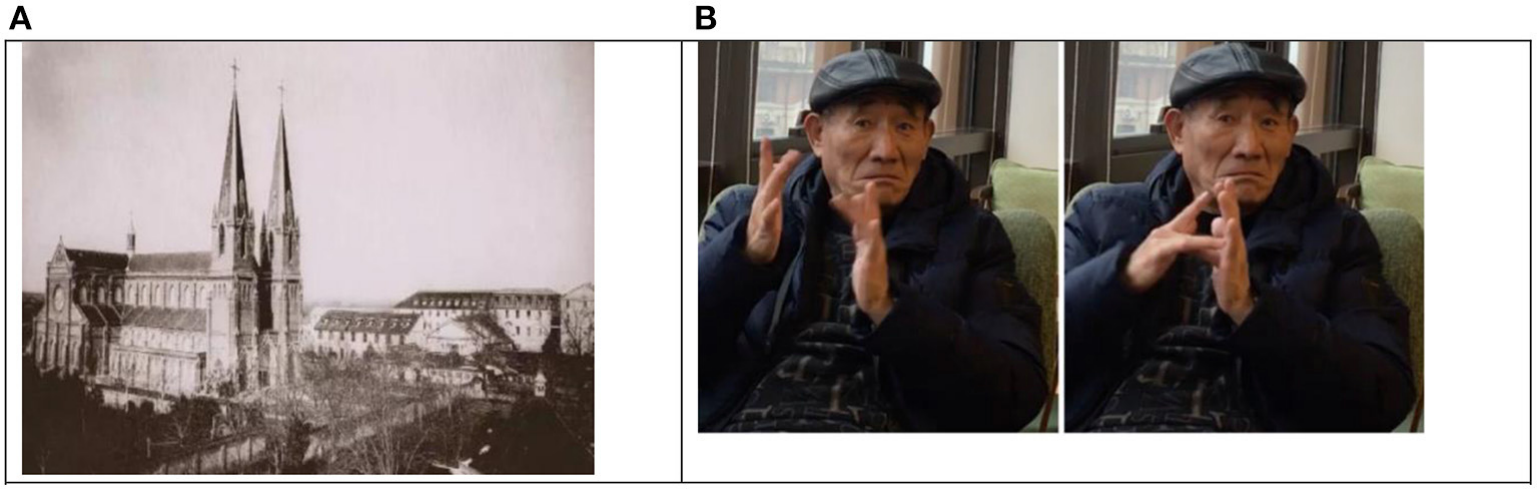
**(A)** Xujiahui Cathedral in 1910 (left). **(B)** Sign place name XUJIAHUI (also a family name).

We can see that the school's situation was hostile to natural sign language since it advocated for oralim with the assistance of French spelling. Communication by sign language was forbidden in school. Though the deaf students learned some basic French signs and mainly French finger-spelling, local sign language soon became their dominant language once they went outside church. As most of the students were deaf females in such a close situation, Xujiahui deaf students' communicative form with an LSF flavor exerted little impact on society. Both Ms. Chen and Ms. Luo could sign some basic words of French sign language, such as *father, mother*, and *Bible*. However, they confided that there was little occasion to use them at all after leaving school. Thus, it is inferred that the influence of French Sign language on early SCSL may be negligible, though there are some traces left in place names, like Xujiahui, which is also identical to the form of Jesus in many Western sign languages. This can be attributed to the fact that Xujiahui Cathedral has long been a landmark in Xujiahui, as shown in Figure 2. In fact now, in SCSL, the sign word JESUS in SCSL is not only the sign for the place name of Xu-jia-hui, but also the sign for Xu, a Chinese family name. Even the interviewees could not tell us where it originated; they have used it as a convention both as a place name and family name since they were children. Maybe it has undergone semantic change to include both meanings. It seems to be specific to Shanghai deaf community. However, we also find that CSL place name *gui-lin(*桂林*)*, the capital city of Guiyang province, a south-western province, use the same form, which is said to be borrowed from Shanghai deaf community. The episode goes like this: When the deaf educators were trained in Shanghai in 1950s, they found a park near Xujiahui called in mandrin guilin park, signed in Shanghai Sign language as jesus park. Then they borrowed it and called their own city jesus. Another example now standardized in CSL is also derived from Xujiahui: gui-hua (桂花,Osmanthus), it is signed as a compound jesus^∧^flower.

### Phase 2 Exemplified by Fry Deaf School

#### Interviewee

Shen Zhuyi, he went to Fry deaf school in 1934 and spent 8 years there, later graduating as a famous deaf artist, and worked as a teacher of painting in a vocational school for the deaf after 1949. He died only a couple of months after our interview. As perhaps the only Fryer Deaf School graduate of his age, he shared with us much information about the use of sign language in the 1930s.

John Fryer died 2 years later, in 1928 in America, his son George became the chancellor. Mr. Wang took charge of the school's daily running, first as director, later as headmaster. He acknowledged the importance of deaf teachers in the school, and many deaf teachers were employed as teachers of key subjects like Chinese (written) and mathematics, though deaf teachers usually did not acquire teaching qualifications. One distinguished deaf student was even employed on his graduation. It offered a range of subjects similar to those in hearing primary schools. Only in a course called speech were Bell's phonic letters used in teaching pronunciation for the lower grades. In other courses for higher grades, sign language seemed to be the primary medium of instruction. Besides, it also offered a course in English, in which American finger-spelling system was used for instruction and assistance. The mixed model practiced by Fryer Deaf must have been quite successful, as around 200 students graduated from 1926 to 1949, and many deaf graduates worked as teachers or even leaders in deaf schools. It contributed to the cultivation of the first deaf students in Shanghai. It made a great contribution to the cultivation of the first Chinese deaf teachers and the development of SCSL.

**Figure 3:**
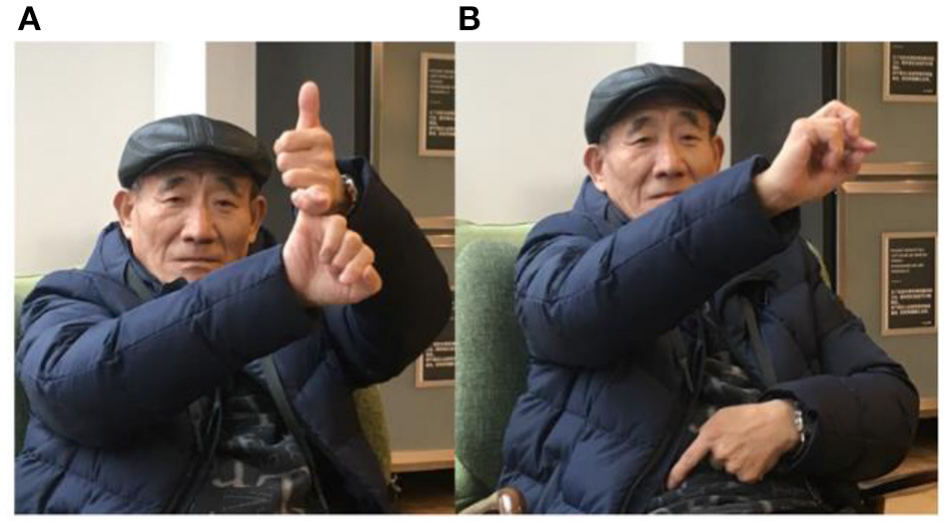
**(A)** SHANGHAI-1(left) and **(B)** SHANGHAI-2 (right).

As American Sign Language (ASL) finger-spelling was used in English classes, we expected some evidence of this in SCSL. However, we could see little direct influence of ASL on SCSL, except for some common signs based on finger-spelling, such as WATER in SCSL, which is signed by shaking the index, middle and ring fingers together, a manual W in ASL. To our perplexity, the place name sign for Shanghai, in SCSL, is probably traceable to British Sign Language (BSL), as shown in Figure 3A. The pinky fingers are hooked together vertically without movement. Since SHANGHI1 (the sign) is a name sign with marked features: two hands, marked handshapes, the similarity between SHANGHAI-1 and finger-spelling S in BSL is not accidental. Moreover, there is a rival variant for Shanghai, SHANGHAI-2, as shown in Figure 3B. It is a one-hand sign, which is widely used for those outside Shanghai as the proper name sign for Shanghai, as well as among some SCSL signers.

**Figure 4:**
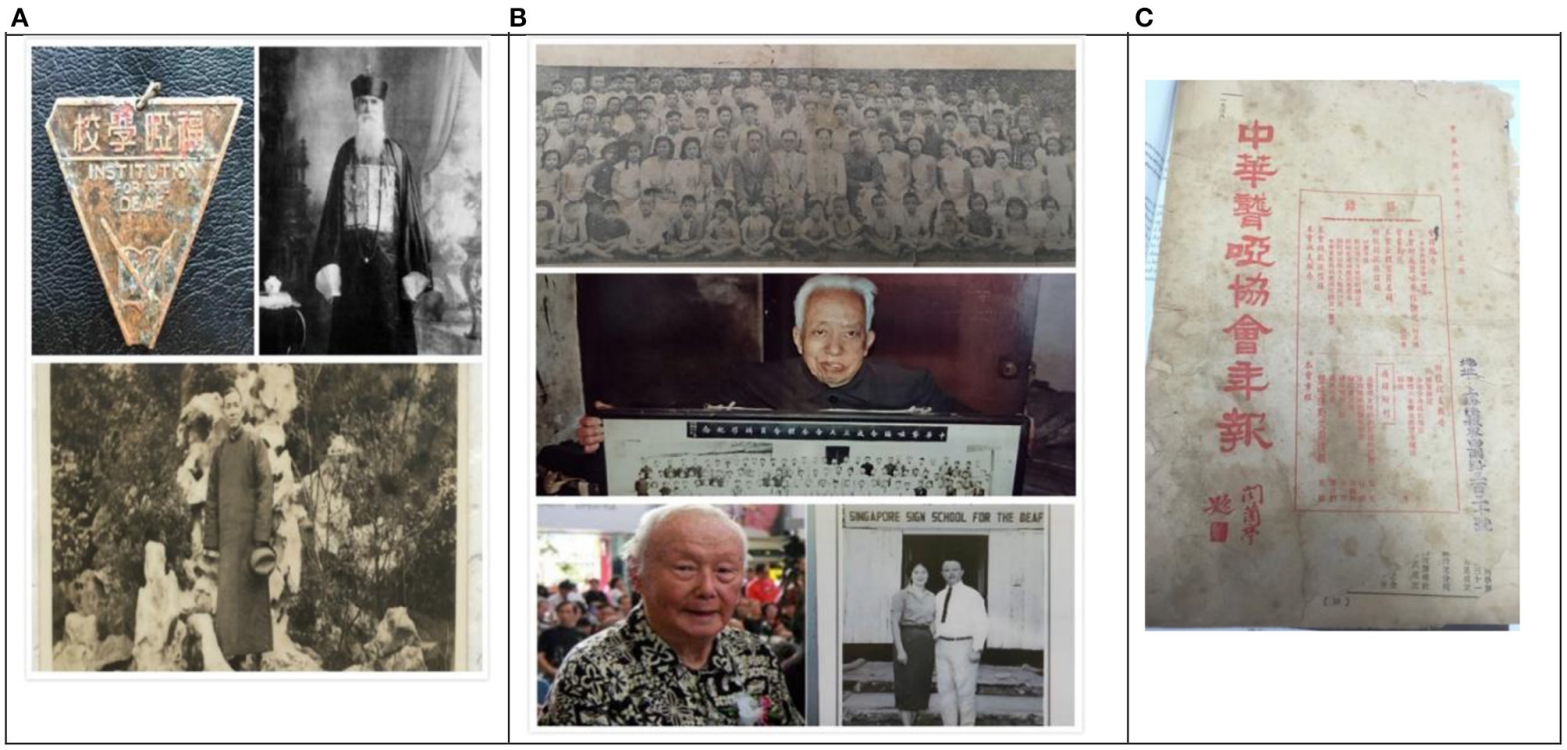
Information about Fryer Deaf, Zhonghua Deaf, and Mr. Peng^a^. **(A)** Badge of Fryer, Mr. Fryer, and Mr. Wang. **(B)** Photos of deaf students and teachers at Zhonghua in 1941, Mr. He, Mr. Peng, and Mr. Peng in the 1950s. **(C)** Annual magazine of the China Deaf Association. ^a^Mr. Peng's interview was conducted by my deaf friend Sheng Huan in Singapore in 2016. When we were planning to arrange another interview in 2018, we were informed of his passing away. The photo was downloaded from an online magazine in Singapore. Available online at: http://www.zaobao.com/keywords/peng-zhu-en (retrieved February 10, 2019).

“The two-hand sign symbolized as Shanghai was invented by Mr. Chuan, a deaf teacher in Fryer Deaf School, who had gone abroad to Britain for his education. He opposed SHANGHAI-2, since it was likely to be mistaken as an obscene gesture as it was signed with the index finger erect and shaking. SHANGHAI-2 was popular in the Zhonghua Deaf School, which was mainly filled by the deaf from other places, seeking refuge in Shanghai as the Sino-Japanese war broke out and forced them to move. The students in Fryer Deaf School accepted SHANGHAI-1, and it was widely acknowledged in the Shanghai deaf community.” (Shen) (Figure 4A).

According to Mr. Shen, SHANGHAI-2 emerged before the production of SHANGHAI-1, the former being an important variant used to refer to Shanghai. However, nowadays, SHANGHAI-1 is the dominant variant among the Shanghai deaf community, while SHANGHAI-2 is widely used outside Shanghai in China. SHANGHAI-2 was probably widely accepted by the deaf of other cities even before the appearance of SHANGHAI-1. After the war, many deaf students went back to their hometown and this may have strengthened the status of SHANGHAI-2. SHANGHAI-1 has won the competition against SHANGHAI-2 among the Shanghai deaf community due to Fryer Deaf School's advocacy and other factors. It is reasonable that SHANGHAI-1 gained the upper hand in this competition due to the high prestige of the Fryer Deaf School within the Shanghai deaf community. ASL is not taught directly, and only a manual alphabet was used in the Fryer Dear School; thus, there is little trace of ASL in SCSL. Though we lacked definite recordings on how sign language was practiced in Group Learning, it was probably similar to that in Fryer Deaf School, as we could see in Shanghai Private Deaf, which stemmed directly from Group Learning, as recorded by Song, who entered the school in 1935, 2 years after its establishment.

“On entering the classroom for the first time, Ms. Shen (a hearing teacher who learned the Cheefoo methodology of speech teaching) used Shouqie (literally “hand pronouncing,” a revised finger-spelling system for teaching the speech of Chinese Mandarin) for teaching our Chinese characters. I was completely at sea and was frustrated because I could not follow this way at all. Then, I consulted a classmate who had been in the school for 5 years. He also shook his head. Later I came to know that none of my classmates really mastered Shouqie. It became only a form, or etiquette, used in the classroom for teaching new Chinese characters. We never used them after class or in our daily lives. In fact, for learning Chinese, we students often translated or even invented signs for Chinese characters. When the teachers saw them, they were also impressed and used the new signs for teaching themselves” (Song, 2000).

Another interviewee, Dai, who spent 3 years in Shanghai Private, then transferred to Fryer Deaf, compared both schools. He believed that Fryer Deaf offered a better learning environment because the deaf teachers in Fryer Deaf were more intelligent and experienced in using sign language for teaching. The quality of deaf staff seemed to become a measure for assessing education quality at that time. On the whole, it is unquestionable that sign language became the main medium of instruction for deaf education in Phase 2. Whether in Fryer or Group learning, the whole environment was very encouraging for sign language to grow.

### Phase 3: Blooming of SCSL During War

Four deaf schools were founded from 1937 to the 1940s: Zhonghua, Deaf Youth, Guangzhen, and Songjiang. They shared several key features: private schools, Chinese deaf educators as founders, sign language as the dominant medium of instruction. The war disrupted the central government's management, and many refugees rushed to Shanghai, including families with deaf children. Meanwhile, the first generation of deaf students graduated from the old deaf school, i.e., Fryer deaf school, and opening private deaf schools became one choice for their careers. For example, the founders of Guangzhen Deaf School were Li Dingqing, who graduated from Fryer Deaf School, and Zhangxun, who taught at Fryer. All the above resulted in the blooming of deaf schools run by the deaf in Shanghai during the 1930s to 1940s. Thus, the founders of Guangzhen deaf schools were students, and later teachers in other early deaf schools in China. As Zhonghua was the most influential, and several interviewees, including its founders, offered detailed information, we will focus on a description of Zhonghua. As Zhonghua is the most representative of this peiord and the most influential deaf school. They also published journal (see Figure 4C), which circulated national wide. Based on the school, they formed the first non-government national deaf associate (中华聋哑协会, Zhonghua deaf-mute associate, Zhonghua is another name for China), please refer to Figure 4B (picture in the middle, Mr. He held up the photo taken when the whole staff attended the first meeting) and Figure 4C (the associate journal), Zhonghua Deaf School (interviewees included Song and Peng).

It was established in 1937, the same year when the all-out anti-Japan war broke out. It represents the deaf schools led by deaf Chinese and was the most influential deaf school from 1937 to 1949. The interviewee Mr. Song (age 96) worked there as a teacher from 1942 to 1946, and was later the headmaster (1946–1950). And the interviewee Mr. Peng worked there as a teaching assistant from 1942 to 1946.

Although there were also hearing teachers, Zhonghua deaf school was led by the deaf, and they forsook the teaching of speech and Shouqie completely, of which they were ignorant themselves. As Shouqie as well as the Cheefoo methodology of speech teaching is similar to oralism that was adopted in Europe and the USA, in a much tone-down way (Lytle et al., 2005).

“We offered 6-year primary education for deaf children in Shanghai, which conformed to the setup of normal hearing schools. Besides, we set up a preparatory grade for younger deaf children, in which we used pictures and signed to them to learn some basic Chinese characters. A 1-year scaffolding course helped the children both in their sign language and Chinese characters. From grade 1 to grade 6, subjects including Chinese literacy (only written Chinese), mathematics, arts, etc. were taught by sign language. We had four deaf teachers and three hearing teachers. All of them were fluent in sign language and taught in sign language. When we met some terms we did not know how to sign, we just created them. Then the sign names, for example, a math term, were adopted naturally and circulated among our school.” Another noticeable trait in Phase 3 is the fluency of sign language for hearing teachers. “All the hearing teachers could sign perfectly. We simply mingled with the students all day and all night. Though the living conditions were very harsh, the children learned in our school quite happily.” (Song)

Mr. Ding also spent 3 years in Zhonghua Deaf school, and he remembered:

“All the teachers could sign very well. We learned from them. Most teachers were deaf. They not only used sign language in the classroom, but also chatted with us, and did story-telling, in sign languages.” (Ding)

Furthermore, these deaf leaders in deaf education were confident and assertive about the role of sign languages rather than Shouqie for deaf education. Song claimed in his memoir: “The essence of deaf education is to teach literacy, thus the ability to read and write Chinese rather than imperfect speech. Sign language is advantageous in helping deaf students in acquiring literacy. Besides, it is beneficial to the communication between students and teachers, forming a harmonious environment in the classroom. In a class with signs as the media of instruction, the students can focus more on the content of the class and be more attentive.” They seemed to be conscious of the power of sign language both as a tool of instruction and a vehicle for cultural inheritance. A student in Song's class recounted: “What impressed me the most were the stories of heroes and anecdotes of Chinese history told by Mr. Song in signs. It was so vivid that all the students gathered around him for one more story.”

#### Phase 3: Start of the Spread of SCSL Overseas

As discussed above, the influence of Zhonghua on the early development of SCSL is significant, in that SCSL was encouraged and regarded as the only medium of instruction, as well as in daily lives, and new signs have been created for educational purposes. Based on their education enterprise, deaf teachers were also leading members of the Zhonghua Deaf Association, which promoted their ideals and their mission that every deaf person could receive education to self-support by publishing their own journals and running exhibitions of deaf artists, etc. It helped the spread of SCSL to foreign deaf communities in Shanghai and other parts of China, like to Hong Kong in the 1940s, when a couple of deaf established a deaf school there (cf. Sze et al., 2013), and Singapore. Many deaf graduated and worked all across China and brought SCSL with them to their localities.

It all started during that time of turmoil, as Sino-Japanese war 1937–1945 was closely followed by civil war (1946–1949). The key figure for SCSL to spread to Singapore was Mr. Peng, a legend (please refer to Figure 4B, the bottom, and cite footnote 7, the obituary from the local newspaper). According to his own account, he was born into a business family on frequent business trips between Hong Kong and his hometown as a child. He first spent 4 years (1937–1941) in a Hong Kong deaf school opened by two Hong Kong speaking Christians, who also followed the model of Cheefoo School, which was the first deaf school in Hong Kong, as it was called Zhen Duo Deaf School (Sun and Liu, 2007, p. 215). As he mentioned in retrospect, deaf students were taught with Shouqie, which simply did not work. He often tutored his classmates via signs. On the breakout of the Pacific war and Japan's invasion of Hong Kong in 1941, he sought refuge in Shanghai and went to Zhonghua as an assistant of He Yulin, the deaf headmaster of Zhonghua, from 1942 to 1946. Later he moved to Singapore, in 1948, and opened the Singapore Chinese Sign School for the Deaf, the first deaf school in Singapore, in 1951. He successfully copied the model of Zhonghua Deaf School and adopted SCSL as the medium of instruction, teaching local deaf students Chinese and other subjects, winning the public and the local government's acclaim. Thus, SCSL was introduced to the Singapore deaf community (Singapore School for the Deaf, 2013).

### Post-phase 3: Transition to a New Era

#### Interviewee

Mr. Zhuang (our model, refer to Figure 2 etc.) went to deaf school from 1949 to 1957, a time of transition. He went to Songjiang Deaf, Yangpu Deaf, and First Deaf School (1955–1957). He gave a comparison of hearing teachers and deaf teachers:

“Only my younger brother and I are deaf in my family. I went to three deaf schools. All those who taught me were deaf teachers with great Shanghai sign language in First Deaf and Songjiang Deaf. At Yangpu Deaf, there were both deaf and hearing teachers. Sometimes hearing teachers only confused us when they mixed speech and signs together, while the deaf teachers gave lectures in sign language, which was much more accessible to us. We learned sign language from them, and we did not create signs ourselves. However, we expressed ourselves more vividly and creatively. I liked old Shanghai Sign Language better. It is like a picture drawn by hands, while the new sign language is obscure.”

### Summary

We have divided into three phases the development of deaf education vis-à-vis roles in sign languages. Table 4 shows the main tendencies in different phases in terms of founders, staff, medium of instruction, medium of daily communication. Founders are classified as two identities: hearing/deaf, Chinese/foreigner; staff fall into three categories: pure hearing, hearing and deaf, deaf-dominant; medium of instruction (class medium) can be classified into the following forms: speech, finger-spelling, sign language. Medium of daily communication (daily medium) refers to what medium is adopted by the students and their teachers to communicate after class in daily life.

**Table 4:** Founders, staff, and medium of instruction in Shanghai deaf schools in different phases.

**Phase**	**Deaf school**	**Founders**	**Hearing status of teaching staff**	**Medium of instruction**
P1	Xujiahui	Hearing, foreigner	Hearing nuns	1. Oralism 2. LSF finger-spelling 3. Basic LSF
P2	Group Learning	Hearing, local laymen	Mainly hearing, only one deaf	Unknown
P2	Wide Love	Hearing, local priests	unknown	Unknown
P2	Fryer	Hearing, foreigner	Half hearing, half deaf	1. SCSL 2. Blackboard writing 3. Finger-spelling
P3–1	Shanghai Private	Hearing, deaf	Hearing-led mixed staff	1. Finger-spelling 2. SCSL
P3–1	Zhonghua	Deaf	Deaf-led, mixed staff	1. SCSL 2. Pictures 3. Writing
P3–1	Deaf Youth	Deaf	Deaf-led, mixed staff	1. SCSL 2. Pictures 3. Writing
P3–2	Guang Zhen	Deaf	Deaf-led, mixed staff	1. SCSL 2. Writing
P3–2	Songjiang	Deaf	Deaf-led, mixed staff	Ditto

Table 4 shows that there is a tendency for deaf teachers and sign languages to be increasingly important.

## Discussion

Three aspects will be discussed based on the analysis above. First, we will explore the timeline for the early development of SCSL. Second, we need to discuss the key factors in the shaping of SCSL during its development. Third is the genetic relationship between the sign language variations used in different schools in Shanghai. We argue that there used to be two variations of SCSL. Besides, we also discuss the impact of SCSL on CSL and its implications for other sign languages.

### Timeline for the Early Development SCSL

While it is difficult to pinpoint the date when the SL variation known as SCSL, deaf elderly signers have indicated that they recognized variations in their sign language use when they were young. Based on their reports, the SCSL variation thrived in schools and was the language used outside of schools when they graduated. We can only trace its development with very limited literature and rare interviews from the senior deaf signers. Considering that, at the turn of Twentieth century, the population of Shanghai concessions increased from 290,000 in 1895 to 610,000 in 1910, it is probable that a small deaf community formed at that time. The majority of deaf were immigrants who followed their families to Shanghai to seek working opportunities. The earliest form of SCSL can be found among them. Though it was the only deaf school in Shanghai at that time, the role of Xujiahui Deaf School in shaping the new sign language is very slight because it was meant to be a Catholic school, and the deaf students were separated from outsiders; and besides, French signs and spelling systems were adopted, which were very difficult for outsiders to pick up due to their complexity and limitations. Maybe the place name of Xujiahui is derived from it. Phase 2 is the key time for SCSL's development, marked by several deaf schools. Sign language became one of the media of instruction in their education and their main medium of communication in their daily lives. With the deaf community expanding and signers from different places in China, to top it all, the opening of public deaf schools, initiated by Group Learning, SCSL may have been undergoing a creolization process in the 1920s. It is assumed that deaf students and teachers gathered in newly-established deaf schools. They had to resort to any resource of local signs or gestures for communication, and to enriched them by daily communicative practice (Nonaka, 2004; Mineiro et al., 2021). These origins of early signs are obscure, we could not find any evidence so far. Based on the distribution of SHANGHAI-1 and SHANGHAI-2, as we found the Fryer alumni prefer SHANGHAI-1 but SHANGHAI-2 is preferred by Group Learning alumni and their descendants, I assume that there were two early key schools that prospered SCSL, one represented by Group Learning and its heir, Shanghai private, the second being Fryer Deaf. Alternatively, Nyst (2012) and Braithwaite (2020) explored the correlation between ecological diversity and linguistic diversity with respect to SL typology. In this view, we won't be troubled by the exact time when SCSL came into being, as long as there is deaf community and effective signing communication, there is language.

1930s saw SCSL to gather momentum and further thrived. Considering the case of Nicaragua Sign Language, it is probable that a sign language can emerge in 20 years as long as deaf schools are established and gatherings and social practices of deaf students are supported. In the early development of SCSL, its conditions seemed to be better than Nicaragua Sign Language as sign language was excluded in the classroom of Nicaragua deaf schools initially (Senghas, 1995), while sign language was not only used in the daily lives of Shanghai deaf students, but became the dominant medium of instruction after Phase 2. As time went by, the deaf in the deaf educational context became ever more self-conscious, and the second generation of deaf students, i.e., those receiving education in Group Learning or Flier Deaf, rose up to take deaf education into their own hands, sign language became the primary medium both in class or out of class in deaf schools. As deaf schools newly opened during phase 3 are more closely connected with the Group learning type, this variant had the upper hand in numbers. However, some deaf teachers in Fryer either participated in the new deaf schools as deaf teachers, or even as founders. The two variants began to merge very quickly in the 1940s. Both types are cornerstones of modern SCSL. Then, it started to spread as the war finished and many deaf returned to their hometown or went overseas, such as the case of Peng in Singapore.

### Key Factors in SCSL's Early Development

Deaf teachers and the implementation of sign language as the medium of instruction are two key factors shaping SCSL. Lexical borrowing could only be found in some frequently used or finger-spelling words like WATER, CLEAN, FINGER-SPELLING, SPEAK, REST, TEACH, etc. (Dai, 1996). Just give an example, Water in SHSL is signed by using the finger alphabet of ASL “W,” which is signed by extending the index, middle and ring finger and shaking horizontally in the air. I assume such a form is borrowed either from ASL or other related sign language. On the other hand, though Xujihui Deaf ushered in the methodology of French deaf education, French sign language (LSF) itself exerted little influence on SCSL, except for some proper name like the place name JESUS, because (1) only some basic French signs were used, and mainly the LSF spelling system was adopted; (2) no deaf French teachers taught at Xujiahui Deaf; (3) Most graduates were girls with a religious background. They were unable or reluctant to use LSF vocabulary or LSF when they left school. Finally, we look at the case of Hong Kong Sign Language. The first deaf school, Hong Kong School for the Deaf was established there in 1935, it followed the model of Cheefoo School, and it has left little impression on Hong Kong Sign Language (HKSL). Instead, another school, Overseas Chinese School for the Deaf and Dumb (OCSD), shaped modern HKSL, because OCSD was opened by a couple of deaf from Shanghai, and sign language was the medium of instruction (Sze et al., 2013). As described, Fryer Deaf School and Group Learning absorbed deaf teachers and adopted sign language as the medium of instruction for practical reasons, even though they made a compromise and also taught speech in the lower grades through finger-spelling. They played pivotal roles in shaping the early SCSL, which further prospered in Phase 3 with the more thorough sign-friendly methodology of the new deaf schools.

### Genetic Relations Among Deaf Schools and the Spread of SCSL

Basically, among all the deaf schools in China, Xujiahui deaf school is unique since it was strictly controlled by French Jesuits, who were mainly influenced by French deaf education and Catholic culture. Though it has also cultivated a great number of deaf students, its influence on SCSL is limited. As we know from Chen and Luo, in the 1940s, most laymen graduates gave up French signs or finger-spellings and adopted SCSL, which echoed the findings of Leeson and Grehan (2004) and LeMaster (2006).

Except for Xujiahui Deaf School, all the other deaf schools are probably genetically related through interaction and the transmission of deaf teacher resources.

**Figure 5:**
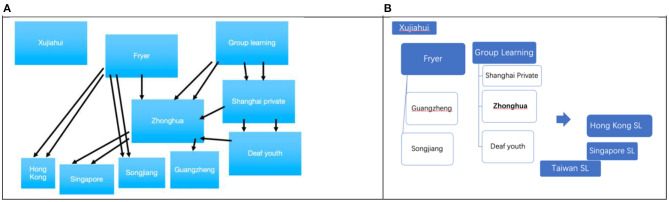
**(A)** Generic relationship within SCSL*. **(B)** SCSL relationship with its neighboring sign languages. *One arrow means deaf students or teachers work in descending school, double arrows show the stronger relation that deaf students or teachers are the founders of the deaf schools to which the arrows are directed.

In Figures 5A,B, Xujiahui Deaf stands alone, while Taiwan SL(TSL) is mainly derived from Japanese Sign Language, some deaf educators from Shanghai moved there to open deaf schools, bringing to current TSL some elements of CSL (Tai and Tsay, 2015). Fryer and Group Learning were two key schools in the early development of SCSL: the former has affected Guangzhen and Songjiang, and exerted some influence on Zhonghua; besides, it gave rise to Hong Kong Sign Language; the latter spawned Shanghai Private, Zhonghua directly, and via them, further affected Deaf Youth. Among them, the Zhonghua style of SCSL was also spread to Singapore by Peng. Though SCSL has influenced HKSL and TSL, it is yet not clear which deaf schools had a direct connection with them, as these two events happened around the same time, i.e., at the end of the 1940s.

### Implications

On investigating the early development of SCSL, one first finds that social context is significant for the appearance of a sign language. The convergence of people and resources in Shanghai from the late Nineteenth to the middle of the Twentieth century provided the conditions for SCSL to grow. Meanwhile, the key to the spread of sign language is deaf teachers. The ideal environment for the early development of a sign language is that represented by Zhonghua Deaf. Second, a sign language seems to grow and become fully fledged in a relatively short time (Meir and Sandler, 2007). In other words, SCSL finished the course in about 20–30 years. When Song (2000), in the early 1930s, attended Deaf Youth, he complained that deaf students had to learn from one another because many new concepts in the textbooks had no equivalents in their shared sign vocabulary; several years later, when he became a teacher at Zhonghua, deaf teachers and students still occasionally created new signs for classroom teaching. This implies that SCSL has been increasingly creolized. However, as the youngest in the older generation, having begun his schooling in 1950, Zhuang told us with confidence that they never created signs themselves. They just learned from their deaf teachers. This suggests that SCSL could play the role of a medium of communication in the classroom quite well in 1950.

Besides, there is a general view that the sign languages of Southern China are similar on the whole (Chen and Gong, 2020); especially, the sign language of Jiangsu province, represented by Nanjing (Capital of China from 1928 to 1949), that of Zhejiang, and that represented by Hangzhou form a triangle that shows much in common in their varieties. This is also attributed to the frequent interactions between the two areas, and SCSL has also impacted on two areas due to their geographical proximity and frequent exchanges. As we can see from the records, many deaf students of Fryer and Zhonghua also moved between Shanghai, Jiangsu (western adjacent province) and Zhejiang (southern adjacent province). In the surge of deaf-leading deaf schools in the 1940s, quite a few deaf in Shanghai deaf schools also moved to Jiangsu and Zhejiang to open deaf schools. As a case in point, Sun Zhuhui, the co-founder of Zhonghua Deaf, also founded deaf schools in Hangzhou and Nanjing. The graduates from Fryer Deaf school, Dai Mu and Shen (interviewees) opened deaf schools in Changzhou, Jiangsu province in 1948 (Dai and Song, 1999). With many deaf people moving back to their hometown and some local Shanghai deaf students working as deaf teachers in other areas in the 1950s, SCSL spread to many areas of China. Shanghai continued to be the leading city in China after 1949, and for deaf education with a strong sign language tradition backed up by a large deaf community, SCSL was and still is one of the most influential variants of Chinese Sign Language (Lin et al., 2009).

## Coda: The Conversion of Private Schools and Their Standardized Names in 1950S

However, the establishment of the PRC in 1949 marked the end of a time when a milestone for deaf education was de facto in a condition of laissez-faire. The government took over all the private schools in the 1950s. Since all of the deaf schools in Shanghai were private schools, some of which were subsidized by charities or other social organizations, the Shanghai government took them over. The majority of them were re-organized, with the deaf teachers remaining, and the deaf culture kept. Based on these old schools, newly-named schools were established in urban areas of Shanghai, as the First, Second, Third and Fourth deaf schools of Shanghai; these four deaf schools made up the leading organizations for Shanghai deaf education as well as being prominent institutions that inherited the heritage of SCSL culture. Interestingly, even the school names were changed, named with ordinal numbers: Deaf 1(the short form of “the First Deaf School of Shanghai,”) Deaf 2 etc. There were four major deaf schools in the downtown of Shanghai, all of which were founded on the old private deaf schools: Deaf 1 succeeded Guang Zhen, Deaf 2 is founded on Fryer Deaf, Deaf 3 inherited Deaf Youth, and Deaf 4 is established on Zhonghua, which merged Shanghai Private before its transform. All the old deaf schools turned from private schools to the government-own deaf schools in 1950s. However, according to the investigation of Mr. Yang, one of my deaf informants, who was a student in 1950s in Third Deaf and became a teacher later in a deaf vocational school, their sign names of these old deaf schools survived, kept in these old alumni. For example, the sign name of Fryer Deaf school is signed by using index finger and middle finger touching at the chin, which is named after the characteristic feature of the founder, Mr. Fryer, who was supposed to have a mole in his chin. Sign name of Guang Zhen was named after the appearance of its founder, who had broken eye. Deaf Youth is named after the logo of Young Men's Christian Association (YMCA)^6^ because YMCA offered it classrooms when Mr. Hu started the school. Thus, the handshape forms a triangle, which is still the sign of contemporary YMCA. Finally, the sign name of Zhonghua deaf is presumed by Yang to derive from the sign name for Confucius: with two hands crossed in a handshaking grip showing the spirit of union.

As a result, the old model of the deaf leading the deaf education took its last breath out of inertia, and was finally engulfed in the new model of all-to-the-nation when national policymakers adopted oralism as the key to deaf education in 1954 (Gao and Gu, 2013). Almost all faded away with the old time.

## Conclusion

**Figure 6:**
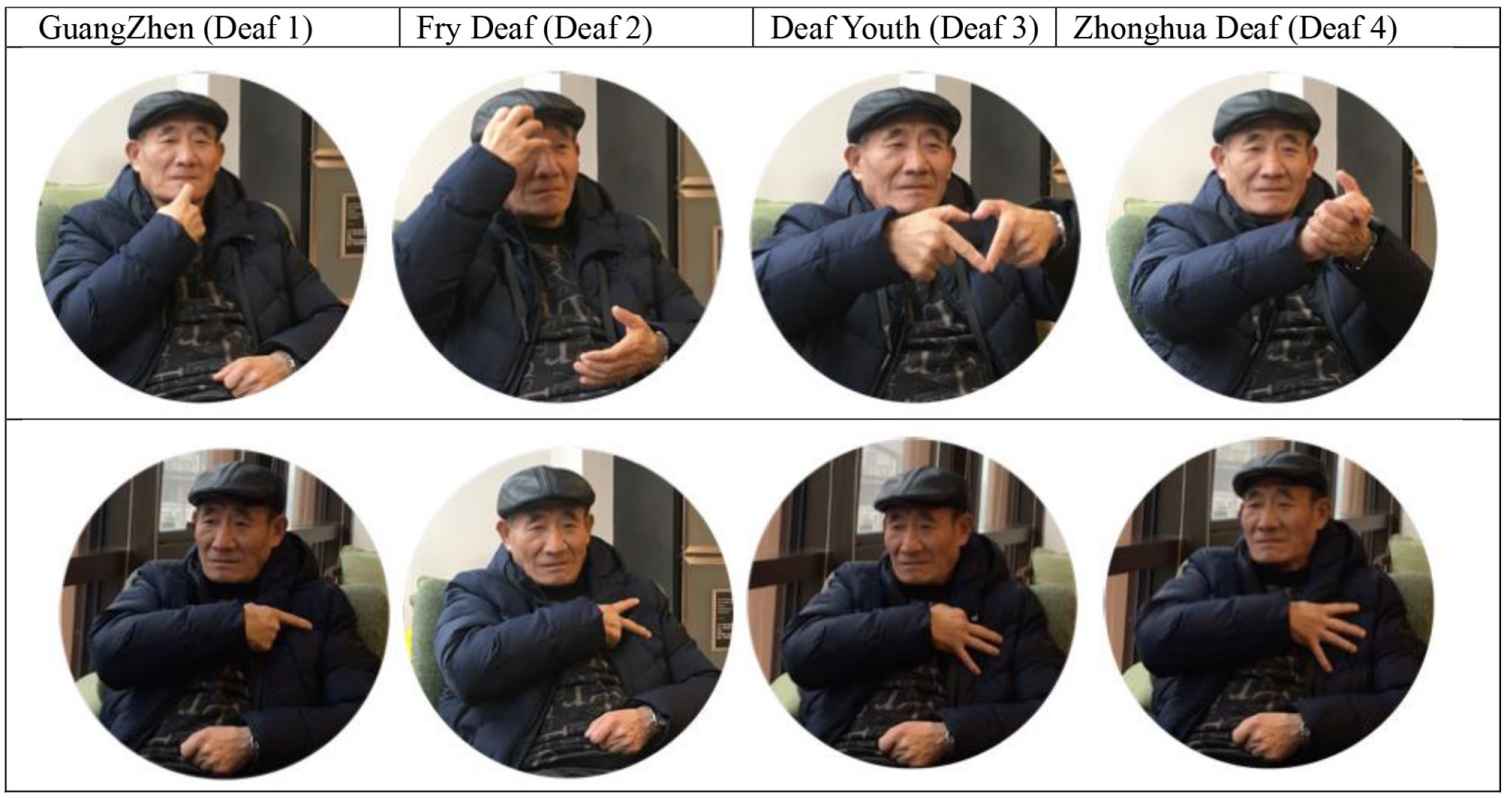
*The sign names of major Shanghai deaf schools before and after conversion (The model is Mr. Zhuang, the first colume is old school signing names and the second colum is the names after conversion). *As shows above, contrastively, all the old sign names are symbolic of the founders or the contributors of the schools while the sign names of their respective successors show the uniformity: numbers of fingers at the left chest suggest of their numbering.

We have traced the early development of SCSL via a historical approach. With a combination of written records and interviews with old deaf subjects, we have found first that SCSL burgeoned at the end of the Nineteenth century (Phase 1), appeared in the 1920s−1930s (Phase 2) with two deaf schools as its backbone: Fryer Deaf and Group Learning, which also formed the two earliest variants within SCSL. Later, a series of deaf schools founded by the deaf, of the deaf, and for the deaf, led by Zhonghua, marked the early development of SCSL as an independent and distinct sign language in at least the later 1930s. Second, two key factors in shaping SCSL were deaf teachers and sign language as the main medium of instruction and communication. Third, in the early history of Shanghai deaf schools, foreigners made a great contribution to the establishment of deaf schools, which were a prerequisite for the early development of SCSL, as shown in the sign names of Fryer Deaf and Deaf Youth, both of which are sign names in the memories of either foreign founders or foreign supporters (cf. Figure 6). However, the influence of foreign sign languages is limited in that the advocacy of oralism as a methodology at that time ruled out foreign deaf teachers' participation in and introduction of foreign sign languages. We could only track some words that may derive from foreign sign languages, as instanced by proper names like XUJIAHUI and SHANGHAI-1, and some common words, water (Dai, 1996), which found their way into Fryer Deaf when English as a foreign language was learned by practicing BSL or ASL, or relevant finger-spellings. It also revealed the multilingual context in the Shanghai deaf community before 1949. We conclude that the development of SCSL suggests that deaf teachers who can play leading roles in deaf schools are very important to the spread of SCSL. This paper on the history of early SCSL shows that it is unique due to its socio-historical complexity with imports of foreign missionaries who ran charity-operated schools who were trained in several Euro-centric SL variations and numerous Shanghai local signers. We also expect more unique characteristic with further research on it as demonstrated in Lin (2019, 2021).

## Data Availability Statement

The raw data supporting the conclusions of this article will be made available by the authors, without undue reservation.

## Ethics Statement

The studies involving human participants were reviewed and approved by Institute of linguistic, Shanghai International Studies University. The patients/participants provided their written informed consent to participate in this study. Written informed consent was obtained from the individual(s) for the publication of any potentially identifiable images or data included in this article.

## Author Contributions

The author confirms being the sole contributor of this work and has approved it for publication.

## Conflict of Interest

The author declares that the research was conducted in the absence of any commercial or financial relationships that could be construed as a potential conflict of interest.

## Footnotes

^1^Shenbao, it was a daily newspaper headquartered in Shanghai, the most widely read newspaper at that time, comparable to the Times in London and the New York Times in New York, in a 1919 issue an advertisement reads that a deaf school has opened, now the enrollment of deaf children has begun….

^2^The boy's name is Lin Jim, of mixed blood. Later, the case made a splash in the society. The public asked for justice and freedom for free love. Shenbao (issues from August 10th, September 8th, October 6th, 1919) also advocated for the innocence of the couple. The court had to submit to popular morals and Lin was released and married his girlfriend, Wu, a hearing girl from a wealthy family. He became famous and a film company even invited the couple to make a movie out of their love story, entitled Dumb Lover.

^3^Zhonghua (literally China) Deaf Mute Association (1937–1952) is the first and only deaf organization so far in China, which is initiated, led and run by the deaf and for the deaf. The founders include He Yulin, Sun Zhuhai et al. Mr. He also founded Zhonghua Deaf School. It was widely supported as a non-governmental organization. Many celebrities including Shanghai mayor Wu Tianchen donated to it as sponsors. It is based in Shanghai and aims to unite all the deaf in China so that they can support one another, especially in seeking education and employment for self-reliance.

^4^As for the names of the deaf schools, some of them have different names in different sources, some of them may not have a formal name at all. I will try to keep them consistent in this paper.

^5^The school is still the only school for the blind in Shanghai. The government took it over in the early 1950s. Mr. Fu Bulan resigned from the presidency and left for America.

^6^I have checked the activities of YCMA, it was very active between 1920s and 1940s, offering a wide range of service for the Chinese youth as well. According to Song (2000), a deaf club was organized by a Chinese Christian Mr. Zhang Qinggen for the deaf people in 1939–1940.

## Appendix 1

Question Items in the Interviews:

Basic educational information

Date of birth, place of birth, sex, age

Which school(s) did you go to? When?

How long did you stay in that or those schools?

Were the teachers hearing or deaf? If you had both deaf and hearing teachers, how did they divide their labor?

Teachers:

Where did the seaf teacher(s) (if any) come from?

What was/were the name(s) of the deaf teacher(s)?

Which medium/media did the deaf teacher(s) use in class?

Was/were the deaf teacher(s) fluent in that/those language(s)?

How well did you understand the lessons given by deaf teachers?

Were the hearing teachers fluent in using the media of instruction (speech, finger-spelling, or natural sign language)?

Would you like hearing teachers to use sign language?

After-class Communication at School

What language did the deaf teachers use when communicating with deaf students after class?

What language did the hearing teachers use when communicating with deaf students after class?

How did students communicate among themselves?

Did students create their own signs?

How did you maintain contact with your schoolmates after you left school?

What did you usually talk about in such gatherings?

What activities did you engage in with your deaf friends?
